# Children’s Experiences of Parental Deaths Due to Suicide, Homicide, Overdose, Alcohol, or Drug Use

**DOI:** 10.1001/jamanetworkopen.2025.31231

**Published:** 2025-09-10

**Authors:** Sean Esteban McCabe, Eric Hulsey, Luisa Kcomt, Rebecca J. Evans-Polce, Glenn Radford, Samuel D. Tennant, Vita V. McCabe

**Affiliations:** 1 Center for the Study of Drugs, Alcohol, Smoking and Health, University of Michigan, Ann Arbor; 2 Survey Research Center, Institute for Social Research, University of Michigan, Ann Arbor; 3 Institute for Healthcare Policy and Innovation, University of Michigan, Ann Arbor; 4 School of Public Health, University of Pittsburgh, Pittsburgh, Pennsylvania; 5 School of Social Work, Wayne State University, Detroit, Michigan; 6 Division for Vital Records and Health Statistics, Michigan Department of Health and Human Services, Lansing; 7 School of Public Health, University of Michigan, Ann Arbor; 8 Addiction Center, Department of Psychiatry, University of Michigan, Ann Arbor

## Abstract

This cohort study investigates parental mortality rates in Michigan, which are above the national average, to consider trends and county-level variability in children who experience parental deaths due to suicide, homicide, overdose, alcohol, or drug use.

## Introduction

Childhood bereavement resulting from parental mortality in the US has increased substantially over the past decade, including a surge in parental deaths from stigmatized causes, which are defined as drug overdose, homicide, suicide, and alcohol-induced or other drug-induced deaths.^1,2,3^ Experiencing the death of a parent during childhood can have a substantial impact on a child’s behavioral, emotional, and mental well-being, especially among children who experience stigmatized deaths and often require specialized bereavement services and treatment.^2,4^ Michigan has parental mortality rates above the national average, which has destabilized many family households.^3,5^ Our objective was to examine statewide trends and county-level variability in Michigan children who experience stigmatized parental deaths to guide the allocation of bereavement services and interventions shown to improve health outcomes.^6^

## Methods

In collaboration with the Division for Vital Records and Health Statistics at the Michigan Department of Health & Human Services, death certificate records were used in this cohort study to identify individuals who died in Michigan between January 2000 and December 2023. Individuals who were also listed on birth certificate records as a biological mother or father were used to identify children born during the period from 1989 to 2023 who appeared on these birth certificates and were collated by year of parental death, year of the child’s birth, and type of parental death on the basis of *International Statistical Classification of Diseases, Tenth Revision* codes, consistent with prior work.^2^ This established a cohort of children aged 17 years or younger who experienced the death of a biological parent (all-cause and stigmatized). The annual count and proportion of children bereaved by stigmatized parental deaths relative to all-cause parental deaths were calculated. Statewide temporal trends were examined using Joinpoint version 5.4.0.0 (National Cancer Institute) regression and a weighted bayesian information criterion method. The percentages of parentally bereaved children from stigmatized death relative to all-cause death were calculated in each Michigan county by dividing the number of children who experienced a stigmatized parental death by the total number of parentally bereaved children between 2000 and 2023 and multiplied by 100. This report follows the STROBE reporting guideline for cohort studies. This research was determined to be exempt from review and the need for informed consent by the University of Michigan’s institutional review board, in accordance with 45 CFR §46.

## Results

**Figure 1.**
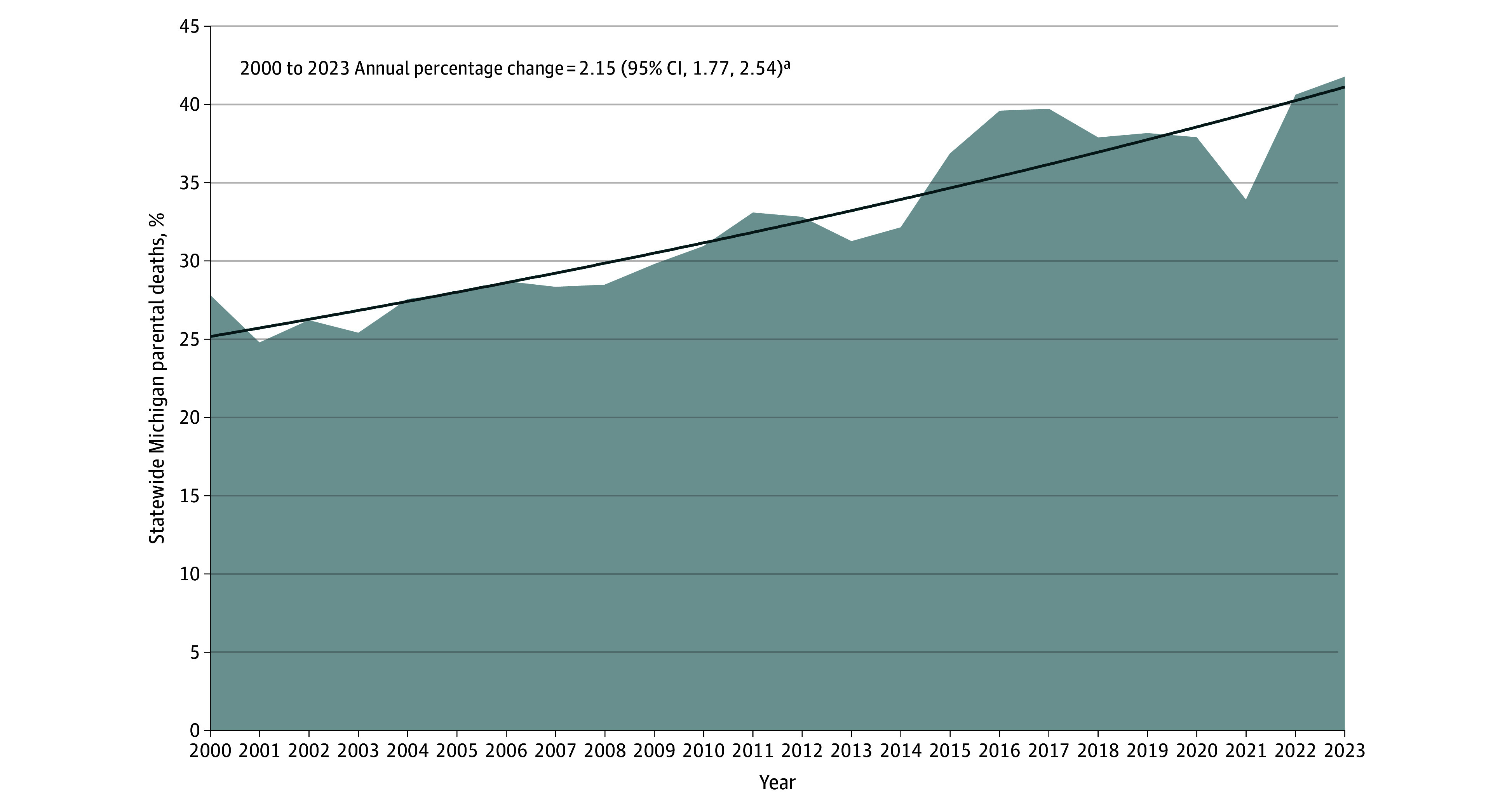
Statewide Percentage of Stigmatized Parental Deaths Relative to All-Cause Parental Deaths in Michigan, 2000 to 2023 The model selection method was weighted bayesian information criterion. The final selected model used 0 joinpoints. Stigmatized deaths were defined as drug overdoses, homicides, suicides, and alcohol- or drug-induced deaths on the basis of the *International Statistical Classification of Diseases, Tenth Revision* codes (see eAppendix in Supplement 1). Data from 2023 are provisional. The statewide percentage of stigmatized parental deaths relative to all-cause parental deaths were 27.82% in 2000, 24.79% in 2001, 26.23% in 2002, 25.42% in 2003, 27.57% in 2004, 27.95% in 2005, 28.70% in 2006, 28.34% in 2007, 28.49% in 2008, 29.80% in 2009, 30.95% in 2010, 33.10% in 2011, 32.82% in 2012, 31.26% in 2013, 32.16% in 2014, 36.87% in 2015, 39.60% in 2016, 39.72% in 2017, 37.90% in 2018, 38.17% in 2019, 37.91% in 2020, 33.92% in 2021, 40.62% in 2022, and 41.77% in 2023. ^a^Indicates that the annual percentage change is significantly different from 0 at the α = .05 level.

**Figure 2.**
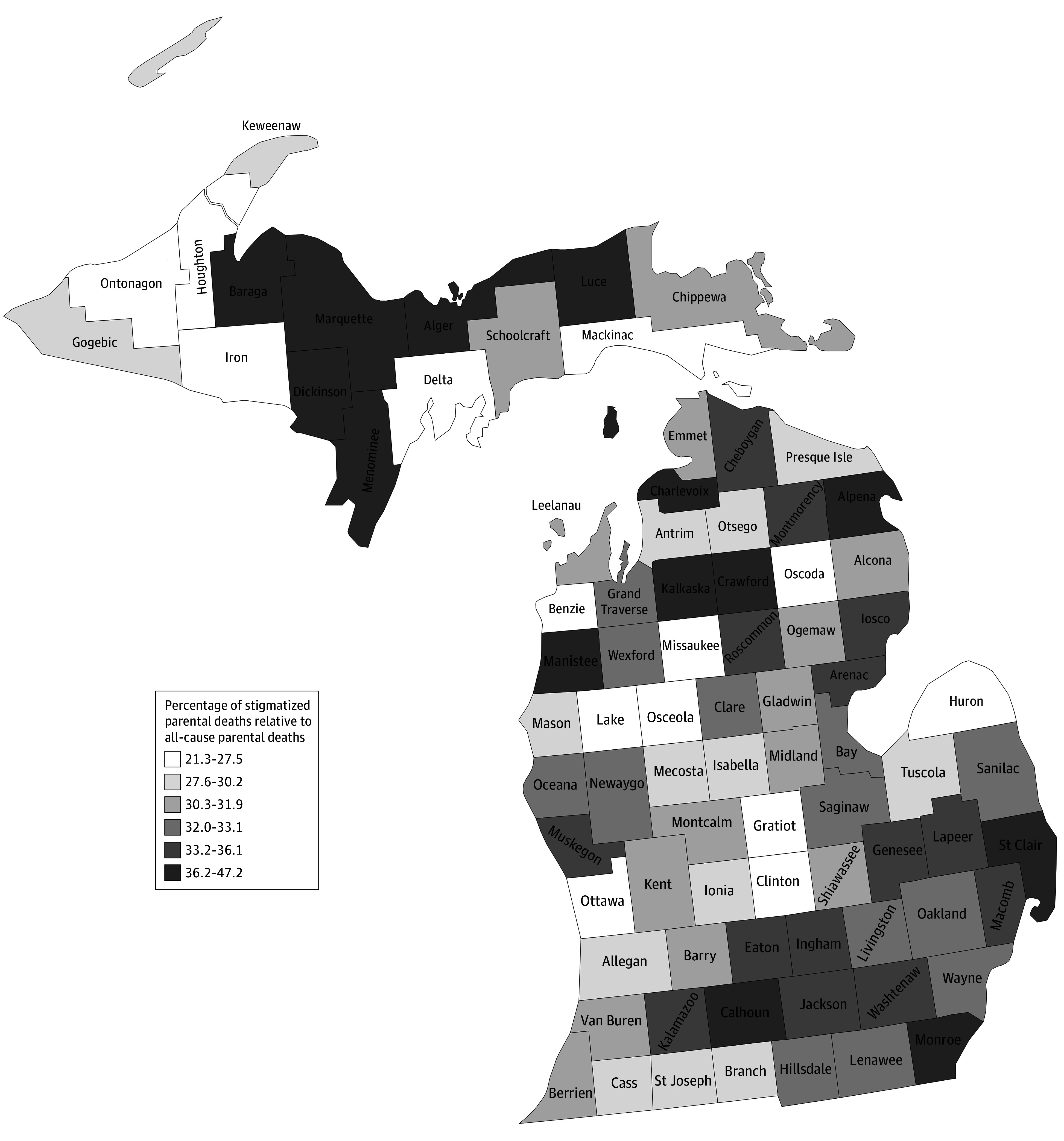
County-Level Percentage of Stigmatized Parental Deaths Relative to All-Cause Parental Deaths in Michigan, 2000 to 2023

Between 2000 and 2023, 115 558 parentally bereaved children from all-cause parental deaths in Michigan were identified, including 38 429 children who experienced stigmatized parental deaths. The annual count and percentage of children with parental bereavement from stigmatized deaths relative to all-cause parentally bereaved children increased significantly between 2000 and 2023 (annual percentage change, 2.15; 95% CI, 1.77, 2.54; *P* < .001) (Figure 1). For instance, stigmatized parental deaths represented 1372 (28.5%) of all parental deaths in 2008 and 2222 (41.8%) of all parental deaths by 2023. At the county level, the percentage of children who experienced stigmatized parental deaths relative to all-cause parental deaths ranged from 21.3% (13 of 61 deaths) to 47.2% (25 of 53 deaths) (Figure 2).

## Discussion

This cohort study found that the number of children bereaved from stigmatized parental deaths has increased and now account for 2 in every 5 parental deaths in Michigan. The increase in children who experienced stigmatized parental deaths is concerning given the increased risks of mental health disorders, child welfare involvement, and criminal justice involvement for children immediately following a parental death.^2,4,5^ There is an urgent need to offer childhood bereavement services for the growing population of at-risk children who experience stigmatized parental deaths.^1,2,3,4,6^ Limitations include those common to vital records research: 2023 data are provisional, not all primary caregivers are biological parents, and fathers have a lower frequency of being listed on birth certificates, resulting in conservative estimates. The increases and county-level variation in children who experienced stigmatized parental deaths offer new information for planning childhood bereavement services. Children and families who are bereaved from a stigmatized death may experience a more complex bereavement process and require a higher level of care. The county-level estimates of children who experience stigmatized parental deaths can inform the allocation of tailored bereavement services in counties with high need.

## Notes

Supplement 1. **eAppendix. ***ICD-10* codes

Supplement 2.
Data Sharing Statement
