# Supplementary material for: Children’s Experiences of Parental Deaths Due to Suicide, Homicide, Overdose, Alcohol, or Drug Use
Source: JAMA Netw Open. 2025 Sep 10;8(9):e2531231. doi: 10.1001/jamanetworkopen.2025.31231 (PMC12423872; doi:10.1001/jamanetworkopen.2025.31231)
Supplement: Supplement 1. — eAppendix. ICD-10 codes [file jamanetwopen-e2531231-s001.pdf]

Supplemental Online Content

McCabe SE, Hulsey E, Kcomt L, et al. Children's experiences of parental deaths due to suicide, homicide, overdose, alcohol, or drug use. *JAMA Netw Open.* 2025;8(9):e2531231. doi:10.1001/jamanetworkopen.2025.31231

eAppendix. *ICD-10* codes

This supplemental material has been provided by the authors to give readers additional information about their work.

© 2025 McCabe SE et al. *JAMA Network Open*.

eAppendix: *ICD-10* codes

Stigmatized deaths were defined drug overdoses, homicides, suicides, alcohol– or drug-induced deaths on the basis of the following *International Statistical Classification of Diseases, Tenth Revision* (*ICD-10*) codes. Drug overdose and drug-induced deaths: *ICD-10* codes D52.1, D59.0, D59.2, D61.1, D64.2, E06.4, E16.0, E23.1, E24.2, E27.3, E66.1, F11.0-F11.5, F11.7-F11.9, F12.0-F12.5, F12.7-F12.9, F13.0-F13.5, F13.7-F13.9, F14.0-F14.5, F14.7-F14.9, F15.0-F15.5, F15.7-F15.9, F16.0-F16.5, F16.7-F16.9, F17.0, F17.3-F17.5, F17.7-F17.9, F18.0-F18.5, F18.7-F18.9, F19.0-F19.5, F19.7-F19.9, G21.1, G24.0, G25.1, G25.4, G25.6, G44.4, G62.0, G72.0, I95.2, J70.2-J70.4, K85.3, L10.5, L27.0-L27.1, M10.2, M32.0, M80.4, M81.4, M83.5, M87.1, R50.2, R78.1-R78.5, X40-X44, X60-X64, X85, and Y10-Y14. Homicide deaths: *ICD-10* codes U01-U02, X85-Y09, Y87.1. Suicide deaths: *ICD-10* codes U03, X60-X84, Y87.0. Alcohol-induced deaths: *ICD-10* codes E24.4, F10, G31.2, G62.1, G72.1, I42.6, K29.2, K70, K86.0, R78.0, X45, X65, Y15.

© 2025 McCabe SE et al. *JAMA Network Open*.
