# Supplementary material for: Children’s Experiences of Parental Deaths Due to Suicide, Homicide, Overdose, Alcohol, or Drug Use
Source: JAMA Netw Open. 2025 Sep 10;8(9):e2531231. doi: 10.1001/jamanetworkopen.2025.31231 (PMC12423872; doi:10.1001/jamanetworkopen.2025.31231)
Supplement: Supplement 2. — Data Sharing Statement [file jamanetwopen-e2531231-s002.pdf]

# Data Sharing Statement

McCabe. Children's Experiences of Parental Deaths Due to Suicide, Homicide, Overdose, Alcohol, or Drug Use. *JAMA Netw Open*. Published September 10, 2025. doi:10.1001/jamanetworkopen.2025.31231

**Data**
**Data available:** Yes
**Data types:** Deidentified participant data
**How to access data:** The data that support the findings of this study are available from the Michigan Department of Health and Human Services (MDHHS), Division for Vital Records & Health Statistics. Restrictions apply to the availability of these data. De-identified or limited datasets are available from the corresponding author with the permission of the MDHHS and a data use agreement.
**When available:** With publication

**Supporting Documents**
**Document types:** None

**Additional Information**
**Who can access the data:** Restrictions apply to the availability of these data. De-identified or limited datasets are available from the corresponding author with the permission of the MDHHS and a data use agreement.
**Types of analyses:** The data will be available for anyone with the permission of the MDHHS and a data use agreement.
**Mechanisms of data availability:** The data will be available for anyone with the permission of the MDHHS and a data use agreement.
